# Differences in baseline cognitive performance between participants with early‐onset and late‐onset Alzheimer's disease: Comparison of LEADS and ADNI

**DOI:** 10.1002/alz.14218

**Published:** 2024-12-23

**Authors:** Dustin B. Hammers, Ani Eloyan, Maryanne Thangarajah, Alexander Taurone, Laurel Beckett, Sujuan Gao, Angelina J. Polsinelli, Kala Kirby, Jeffrey L. Dage, Kelly Nudelman, Paul Aisen, Rema Reman, Renaud La Joie, Julien Lagarde, Alireza Atri, David Clark, Gregory S. Day, Ranjan Duara, Neill R. Graff‐Radford, Lawrence S. Honig, David T. Jones, Joseph C. Masdeu, Mario F. Mendez, Kyle Womack, Erik Musiek, Chiadi U. Onyike, Meghan Riddle, Ian Grant, Emily Rogalski, Erik C. B. Johnson, Steven Salloway, Sharon J. Sha, Raymond Scott Turner, Thomas S. Wingo, David A. Wolk, Maria C. Carrillo, Bradford C. Dickerson, Gil D. Rabinovici, Liana G. Apostolova, the LEADS Consortium 1 for the Alzheimer's Disease Neuroimaging Initiative

**Affiliations:** ^1^ Department of Neurology Indiana University School of Medicine Indianapolis Indiana USA; ^2^ Department of Biostatistics Center for Statistical Sciences Brown University Providence Rhode Island USA; ^3^ Department of Public Health Sciences University of California—Davis Davis California USA; ^4^ Department of Biostatistics Indiana University School of Medicine Indianapolis Indiana USA; ^5^ Department of Medical and Molecular Genetics Indiana University School of Medicine Indianapolis Indiana USA; ^6^ Alzheimer's Therapeutic Research Institute University of Southern California San Diego California USA; ^7^ Department of Neurology University of California—San Francisco San Francisco California USA; ^8^ Banner Sun Health Research Institute Sun City Arizona USA; ^9^ Department of Neurology Mayo Clinic Jacksonville Florida USA; ^10^ Wien Center for Alzheimer's Disease and Memory Disorders, Mount Sinai Medical Center Miami Florida USA; ^11^ Taub Institute and Department of Neurology Columbia University Irving Medical Center New York New York USA; ^12^ Department of Neurology Mayo Clinic Rochester Minnesota USA; ^13^ Nantz National Alzheimer Center Houston Methodist and Weill Cornell Medicine Houston Texas USA; ^14^ Department of Neurology David Geffen School of Medicine at UCLA Los Angeles California USA; ^15^ Department of Neurology Washington University in St. Louis St. Louis Missouri USA; ^16^ Department of Psychiatry and Behavioral Sciences Johns Hopkins University School of Medicine Baltimore Maryland USA; ^17^ Department of Neurology Alpert Medical School Brown University Providence Rhode Island USA; ^18^ Department of Psychiatry and Behavioral Sciences Mesulam Center for Cognitive Neurology and Alzheimer's Disease Feinberg School of Medicine Northwestern University Chicago Illinois USA; ^19^ Healthy Aging & Alzheimer's Research Care Center Department of Neurology University of Chicago Chicago Illinois USA; ^20^ Department of Neurology Emory University School of Medicine Atlanta Georgia USA; ^21^ Department of Neurology & Neurological Sciences Stanford University Palo Alto California USA; ^22^ Department of Neurology Georgetown University Washington D.C. USA; ^23^ Department of Neurology UC Davis Alzheimer's Disease Research Center University of California—Davis Davis California USA; ^24^ Department of Neurology Perelman School of Medicine University of Pennsylvania Philadelphia Pennsylvania USA; ^25^ Medical & Scientific Relations Division Alzheimer's Association Chicago Illinois USA; ^26^ Department of Neurology Massachusetts General Hospital and Harvard Medical School Boston Massachusetts USA; ^27^ Department of Radiology & Biomedical Imaging University of California—San Francisco San Francisco California USA; ^28^ Department of Radiology and Imaging Sciences Center for Neuroimaging Indiana University School of Medicine Indianapolis Indianapolis Indiana USA

**Keywords:** Alzheimer's disease, amnestic, early‐onset, late‐onset, non‐amnestic

## Abstract

**INTRODUCTION:**

Early‐onset Alzheimer's disease (EOAD) and late‐onset Alzheimer's disease (LOAD) share similar amyloid etiology, but evidence from smaller‐scale studies suggests that they manifest differently clinically. Current analyses sought to contrast the cognitive profiles of EOAD and LOAD.

**METHODS:**

*Z‐*score cognitive‐domain composites for 311 amyloid‐positive sporadic EOAD and 314 amyloid‐positive LOAD participants were calculated from baseline data from age‐appropriate control cohorts. *Z‐*score composites were compared between AD groups for each domain.

**RESULTS:**

After controlling for cognitive status, EOAD displayed worse visuospatial, executive functioning, and processing speed/attention skills relative to LOAD, and LOAD displayed worse language, episodic immediate memory, and episodic delayed memory.

**DISCUSSION:**

Sporadic EOAD possesses distinct cognitive profiles relative to LOAD. Clinicians should be alert for non‐amnestic impairments in younger patients to ensure proper identification and intervention using disease‐modifying treatments.

**Highlights:**

- Both early‐onset Alzheimer's disease (EOAD) and late‐onset Alzheimer's disease (LOAD) participants displayed widespread cognitive impairments relative to their same‐aged peers.
- Cognitive impairments were more severe for EOAD than for LOAD participants in visuospatial and executive domains.
- Memory and language impairments were more severe for LOAD than for EOAD participants
- Results were comparable after removing clinical phenotypes of posterior cortical atrophy (PCA), primary progressive aphasia (lv‐PPA), and frontal‐variant AD.

## 1 INTRODUCTION

Although early‐onset Alzheimer's disease (EOAD) and late‐onset Alzheimer's disease (LOAD) are associated with amyloid beta (Aβ) and tau deposition,^1^, ^2^, ^3^ preliminary evidence suggests that they are not identical conditions differing only by age. EOAD—presenting between 40 and 64 years of age^4^—tends to manifest with less hippocampal/medial temporal atrophy and degradation than is the hallmark for LOAD.^1^ Instead, the condition is often associated with greater parietal and overall cortical atrophy,^5^ posterior cingulate and parietal white‐matter degradation,^6^ and parietal lobe hypometabolism.^7^ The clinical presentations between the two also appear to be distinct. EOAD presents with greater symptom severity and aggressive disease course,^8^, ^9^ and it has been suggested that EOAD is less associated with the traditional amnestic predominance of LOAD.^10^, ^11^, ^12^, ^13^, ^14^, ^15^ Clinical phenotypes of frontal‐variant AD, posterior cortical atrophy (PCA), and logopenic variant primary progressive aphasia (lv‐PPA) are reported to be more common in early‐onset disease.^16^, ^17^, ^18^

Most research on EOAD to date reflects relatively small sample sizes and single‐site cohorts.^9^, ^12^, ^13^, ^14^, ^15^ The Longitudinal Early‐Onset Alzheimer's Disease Study ([LEADS]; *National Institute on Aging [NIA] R56057195, NIA U016057195*)^19^ was launched in 2018, with the primary goal of providing a comprehensive understanding of the clinical and pathophysiologic manifestations of sporadic EOAD in a sample of 600 cognitively impaired participants from 18 sites across the United States. Recently findings from LEADS at the midpoint of data collection have shown that sporadic EOAD possesses a unique atrophy signature,^20^ advanced tau binding at baseline (via both imaging^2^ and cerebrospinal fluid analysis^21^) that is associated with white‐matter hyperintensity burden,^22^ high rates of neuropsychiatric symptoms,^23^ and rates of apolipoprotein (*APOE*) ε4 carriers that were generally lower than findings in LOAD.^24^, ^25^, ^26^ In addition, LEADS observed a low frequency of pathogenic variants in known causative mutations including in the amyloid precursor protein (*APP*), presenilin‐1 (*PSEN1*), and presenilin‐2 (*PSEN2*) for familial AD; granulin precursor aka progranulin (*GRN*); microtubule‐associated protein tau (*MAPT*); or chromosome 9 open reading frame 72 (*C9ORF72*) for familial frontotemporal dementia,^25^ associated with recruitment goals of excluding those with strong family history of EOAD. LEADS has similarly advanced our knowledge of the cognitive manifestations of EOAD, showing that participants with amyloid‐positive EOAD possess worse cognitive performance at baseline on initial learning processes^27^, ^28^ and domains of episodic memory, executive functioning, and speed/attention than patients presenting with early‐onset cognitive impairment but found to be amyloid negative.^11^

The aim of the current study is, therefore, to build upon this work by comparing the cognitive profiles of participants with EOAD and those with LOAD directly. The Alzheimer's Disease Neuroimaging Initiative (ADNI)^29^, ^30^ cohort is an ideal LOAD comparison group to LEADS because it is also a longitudinal multicenter study of sporadic late‐onset AD, albeit one focused on individuals who develop AD at mostly older rather than younger ages. LEADS and ADNI protocols were harmonized to ensure compatibility of cognitive, biomarker, and neuroimaging assessments. It is important to note that both ADNI and LEADS include cognitively normal (CN) participants in their samples, which permit age‐normative referencing across cohorts. Given previous research, it was hypothesized that EOAD participants would present with worse cognitive performance than LOAD participants at baseline in non‐memory domains, even after controlling for potentially confounding clinical variables like severity cognitive impairment *APOE ε*4 status. Relatedly, it was hypothesized that greater memory impairments would be observed in LOAD participants. Should these hypotheses be correct, they would provide insights into the unique cognitive manifestation of sporadic EOAD. They would also highlight the importance of identifying non‐amnestic change early in biomarker‐confirmed younger populations, so that these individuals are not overlooked early in the disease process for recently‐approved disease‐modifying treatments.

## 2 METHODS

### 2.1 Participants

#### 2.1.1 LEADS participants

As of January 31, 2024, a total of 311 EOAD and 94 younger CN participants (referred hereafter as LEADS‐CN) have been enrolled in LEADS with data that have been quality controlled. Full inclusion/exclusion criteria have been reviewed elsewhere.^19^ Briefly, all participants were between 40 and 64 years of age at baseline, in good general health, and absent other neurological or psychiatric disorder. In addition, they were fluent in English and had the availability of a study partner to serve as a knowledgeable informant. Participants with mutations in *APP*, *PSEN1*, *PSEN2*, *MAPT*, *C9ORF72*, or *GRN*—or family history of multiple first‐degree relatives with EOAD—were excluded, as were participants receiving experimental drug treatment for AD. Diagnosis of EOAD or CN was assigned via consensus during formal review among cognitive neurologists, neuropsychologists, and geriatric psychiatrists. All EOAD participants presented with a possible AD phenotype (e.g., progressive cognitive decline in the absence of non‐neurodegenerative contributors or core clinical criteria for non‐AD dementia), met the age range for “early‐onset dementia”, were amyloid positive in this study (as defined below), and were classified as mild cognitive impairment (MCI) or mild dementia. Consideration of non‐amnestic phenotypes was based on previous established criteria (^31^, ^32^ for *PCA* and lv‐*PPA*, respectively), or the current state of research for phenotypes lacking established criteria (i.e., frontal‐variant AD).^33^ EOAD participants possessed a Clinical Dementia Rating (CDR)^34^ scale global score of 0.5 or 1.0 at baseline, whereas LEADS‐CN participants possessed a Mini‐Mental State Examination (MMSE)^35^ score of ≥ 24 and a CDR global score of 0. LEADS‐CN participants met the age range (40‐64 years) and were included regardless of amyloid status. A central‐institutional review board (IRB) at Indiana University School of Medicine approved and oversaw all study procedures, and formal informed consent was obtained in writing from study participants or their legally authorized representatives.

**RESEARCH IN CONTEXT**

1. **Systematic review**: Traditional PubMed searches into the limited literature on sporadic early‐onset Alzheimer's disease (EOAD) versus late‐onset Alzheimer's disease (LOAD) were utilized to review known information about cognitive profiles of each condition. Although limited comparisons between EOAD and LOAD have been conducted, sample sizes tend to be small and disease severity is rarely considered.
2. **Interpretation**: In a sample of 311 EOAD and 314 LOAD participants, results showed that EOAD displayed greater cognitive impairments than LOAD in visuospatial and executive domains, whereas LOAD displayed greater impairments in amnestic and language domains.
3. **Future directions**: Future investigation will explore comparisons of longitudinal cognitive trajectories between EOAD and LOAD.

#### 2.1.2 ADNI participants

All LOAD participant data in the present study were obtained from ADNI's multicenter longitudinal study. The reader is referred to the ADNI website (http://adni.loni.usc.edu) for a thorough review of study resources and details on accessing the publicly available data. As has been described elsewhere, ADNI was launched in 2003 as a public–private partnership, led by Principal Investigator Michael W. Weiner, MD. The main goal of ADNI has been to test whether serial magnetic resonance imaging (MRI), positron emission tomography (PET), other biological markers, and clinical and neuropsychological assessment can be combined to measure the progression of MCI and dementia due to LOAD. Please see www.adni‐info.org for up‐to‐date information. IRB approval has been obtained locally for each of the multicenter sites, and written informed consent was obtained from study participants or their authorized representatives.

The main aims of LEADS include comparing neuroimaging‐based AD biomarkers between EOAD and LOAD samples. Therefore, in an effort to harmonize samples across several upcoming projects and analyses, the current ADNI sample comprised participants receiving either ADNI‐2 or ADNI‐3 protocols.^29^, ^30^ Consequently, baseline cognitive data were available for 314 amyloid‐positive LOAD participants (as defined below) and 354 older CN participants (referred hereafter as ADNI‐CN), with baseline visits occurring between February 2011 and January 2020. Although ADNI includes individuals 55 to 90 years of age at baseline, only those participants ≥65 years of age were included in the current study to meet LOAD criteria. Additional ADNI inclusion criteria include having at least 6 years of education, absence of significant head trauma/depression/neurologic disease, having a reliable study partner, being stable on permitted medications, and fluency in either English or Spanish. ADNI diagnostic classification criteria have been described thoroughly in the literature^29^; briefly, they include specific performances on Logical Memory from the Wechsler Memory Scale–Revised (WMS‐R),^36^ the MMSE, and the CDR. To be selected for the current analyses, ADNI participants must have had MRI and amyloid PET data and (to be consistent with LEADS) a CDR global score of 0 at baseline for ADNI‐CN and 0.5 or 1.0 at baseline for LOAD.

### 2.2 Procedures

EOAD participants underwent standard study procedures for LEADS,^19^ and LOAD participants took part in study procedures from either ADNI‐2 or ADNI‐3.^29^, ^30^

#### 2.2.1 LEADS procedures

In LEADS, participants received a standardized baseline clinical assessment, which incorporated medical and family history, medication review, and medical/neurological examinations. Cognitive assessment was undertaken using the National Alzheimer's Coordinating Center's (NACC) Uniform Data Set (UDS) 3.0,^37^ and several LEADS‐specific measures that were also used in ADNI—including the MMSE, Rey Auditory Verbal Learning Test (RAVLT),^38^ and Alzheimer's Disease Assessment Scale–Cognitive Subscale (ADAS‐Cog).^39^ PET was conducted to assess for Aβ deposition (^18^F‐Florbetaben) and to confirm classification of EOAD. Additional cognitive measures, measurement of functioning, blood draw, and brain imaging were administered in LEADS that are not germane to the current comparisons.

#### 2.2.2 ADNI procedures

All ADNI participants underwent an extensive clinical and neuropsychological battery at a baseline visit upon their enrollment, which as described above matched relevant tests in the LEADS neuropsychological battery. Participants in the current study also underwent amyloid‐PET imaging using either the ^18^F‐Florbetapir or ^18^F‐Florbetaben ligand, depending on the ADNI protocol.^29^, ^30^

### 2.3 Determination of Aβ positivity

In cognitively impaired participants across both LEADS and ADNI, PET was used to identify amyloid‐positive cases via a process that was developed originally in LEADS and described previously in detail.^2^ The same approach was applied to ADNI scans, with a slight modification to accommodate for different radiotracers; all LEADS scans were performed with ^18^F‐Florbetaben, whereas ADNI participants received either ^18^F‐Florbetaben (*n *= 214) or ^18^F‐Florbetapir (*n *= 454). All LEADS and ADNI scans were analyzed centrally at University of California San Francisco (UCSF) by combining expert visual read and amyloid‐PET quantification to maximize confidence in the final scan interpretation.

Briefly, each amyloid‐PET scan was interpreted visually by a certified clinician according to published and U.S. Food and Drug Administration (FDA)–approved criteria,^40^, ^41^ without access to the participant's clinical information or PET quantification. In parallel, scans were pre‐processed using a PET‐only pipeline (see Cho et al.^2^ for details), which resulted in a global standardized uptake value ratio (SUVR) quantification with tracer‐specific positivity threshold values as follows: 1.18 for ^18^F‐Florbetaben and 1.15 for ^18^F‐Florbetapir. If visual interpretation and quantification‐based classification were incongruent (e.g., visually read as positive but ^18^F‐Florbetapir SUVR value <1.15, or vice versa), another visual read was performed by a second reader who was blinded to the previous read and quantification. Note that, even in cases with congruent visual read/quantification, the original reader could ask for a second opinion in case of an ambiguous or particularly challenging scan. This second read was used as a tie breaker to determine amyloid status.

### 2.4 Cognitive composites

The following cognitive measures were administered in both LEADS and ADNI: Montreal Cognitive Assessment (MoCA),^42^ RAVLT, ADAS‐Cog, Trail Making Test Parts A and B,^43^ and Animal Fluency (semantic fluency).^44^ The use of crosswalks^45^ was required for comparing story memory (Craft Story 21 Memory Test^46^ for LEADS and Logical Memory for ADNI) and confrontation naming (Multilingual Naming Test [MINT]^47^ for LEADS and MINT or Boston Naming Test^48^ for ADNI, depending on the protocol) between the two studies. Because these tests are familiar to most dementia clinicians and researchers, they will not be described here. Cognitive composites were calculated from subtests of these measures, resulting in domain composites of Episodic Immediate Memory, Episodic Delayed Memory, Language, Processing Speed/Attention, Visuospatial, and Executive skills (see Table 1 for a listing of specific variables per domain). Each domain score was calculated separately for LEADS and ADNI participants from age‐appropriate control cohorts. Normality of individual tests was assessed using Shapiro‐Wilk test, and was found to be violated; consequently the median was incorporated into the composite analyses instead of the mean. Residuals from the respective CN group were calculated by controlling for education for each clinical score at baseline. More specifically, for the calculation of the EOAD domain composite values, residuals from the younger LEADS‐CN participants were used, and for the calculation of the LOAD domain composites, residuals from the older ADNI‐CN participants were incorporated. Following the creation of these residuals, each clinical variable was centered using the median absolute difference (MAD) (or mean absolute difference, if needed), as follows; *MAD *= median*_i_* |*x_i_*−*x̄*|.^49^, ^50^ The MAD was subtracted for each variable and standardized, approximating the standard deviation standardization to calculate *robust Z‐*scores, *Z‐*score = (*X*−*Median*)/(1.486∗*MAD*). If *MAD* was equal to zero, the *MeanAD* was used in the scale estimate to calculate the *robust Z‐*scores, *Z‐score *= (*X*−*Median*)/(1.253∗*MeanAD*). The *robust Z‐*scores were grouped by cognitive domains and for each participant the average was taken for each domain for the composite value.

**TABLE 1:** Test measures comprising the cognitive composites per domain.

Domain	Measures		Crosswalk needed
	LEADS	ADNI	
Processing Speed/Attention	Trail Making Test Part A	Trail Making Test Part A	No
	Number Span Forward	Number Span Forward	No
Episodic Delayed Memory	RAVLT Delayed Recall	RAVLT Delayed Recall	No
	Craft Story 21 Memory Test—Delayed Paraphrase Recall	Logical Memory II	Yes
Language	MINT	MINT or Boston Naming Test	Yes
	Animal Fluency	Animal Fluency	No
	ADAS‐Cog (Word Recognition, Naming Objects and Fingers, Commands)	ADAS‐Cog (Word Recognition, Naming Objects and Fingers, Commands)	No
Visuospatial Skill	MoCA Clock (Hands, Numbers)	MoCA Clock (Hands, Numbers)	No
	MoCA Cube	MoCA Cube	No
Executive Functioning	Trail Making Test Part B	Trail Making Test Part B	No
Episodic Immediate Memory	RAVLT Total Recall	RAVLT Total Recall	No
	Craft Story 21 Memory Test—Immediate Paraphrase Recall	Logical Memory I	Yes

Abbreviations: ADAS‐Cog, Alzheimer's Disease Assessment Scale–Cognitive Scale; ADNI, Alzheimer's Disease Neuroimaging Initiative; LEADS, Longitudinal Early‐onset Alzheimer's Disease Study; MINT, Multi‐lingual Naming Test; MoCA, Montreal Cognitive Assessment; RAVLT, Rey Auditory Verbal Learning Test.

### 2.5 Data analysis

Demographic analyses were performed using *t‐*tests for continuous and chi square analysis for categorical variables. Initial examination of the cognitive composites between EOAD and LOAD groups was conducted using non‐parametric Kruskal‐Wallis rank sum tests without covariation to appreciate differences without accounting for the severity of the cohorts. For the primary analyses, however, where we sought to examine domain‐specific deficits above and beyond global cognitive severity, linear regression analyses were used to compare performance of groups on the cognitive composites, after accounting for sex, cognitive severity (MMSE), and *APOE ε*4 status. Finally, to ensure that the results were not driven by different rates of amnestic versus non‐amnestic predominance between the EOAD and LOAD cohorts, supplementary sensitivity analyses were conducted where the primary analyses were re‐run after removing the EOAD cases classified as having clinical phenotypes of frontal‐variant AD, lv‐PPA, or PCA; these variants are not present in ADNI because memory loss is part of ADNI inclusion criteria. Therefore, this step was not conducted for LOAD cases. Measures of effect size were expressed as Cohen's *d* (*t‐*tests), partial eta squared (linear regression analyses), and Cohen's *w* values (chi‐square analyses). *P*‐values were adjusted using false discovery rate to account for multiple comparisons, with subsequent significance being set at *p *< 0.05.

## 3 RESULTS

Table 2 displays demographic, clinical, and biomarker characteristics of participants in LEADS and ADNI. When compared to the LEADS‐CN group, the EOAD group was younger, had fewer years of education, and had a lower racial/ethnic diversity (*p’*s < 0.001, Cohen's *d’*s = 0.51 to 0.54, Cohen's *w *= 0.25). Rates of *APOE* ε4 carriers were higher in EOAD than LEADS‐CN (*p *= 0.02, *w *= 0.12). The LOAD group was older and less educated than the ADNI‐CN group, and had a lower proportion of women (*p’*s = 0.001 to 0.002, *d’*s = 0.35 to 0.55, *w *= 0.12). *APOE* ε4 carrier rates were also higher in LOAD than ADNI‐CN (*p *< 0.001, *w *= 0.34). Both impairment groups performed worse than their respective CN groups on the MMSE (*p’*s < 0.001, *d’*s = 1.61 to 1.67). When comparing between impairment groups, no differences were observed for sex or racial/ ethnic diversity (*p’*s > 0.05), although the EOAD group has lower education levels (*p *= 0.02, *d *= 0.18). By design, the EOAD group was significantly younger than the LOAD group (*p *< 0.001, *d *= 3.73). The EOAD group was more severely impaired (lower scores on the MMSE *[p *< 0.001, *d *= 0.85], higher CDR global scores [*p *= 0.004, *w *= 0.12], and higher rates of participants classified as dementia [*p *< 0.001, *w *= 0.28]) than the LOAD group, and possessed lower rates of *APOE* ε4 carriers (*p *= 0.007, *w *= 0.11).

**TABLE 2:** Baseline demographic characteristics of the EOAD, LOAD, and CN groups.

	EOAD	LEADS‐CN	LOAD	ADNI‐CN	EOAD—LOAD *p* Value (effect size)
*N*	311	94	314	354	
Age in years^a^, ^c^	58.59 (3.9)	56.16 (6.0)	77.25 (5.9)	73.92 (6.4)	< 0.001 (3.73)
Sex (% female)^c^	52.4%	62.8%	43.9%	56.2%	0.03 (0.09)
Non‐White/Hispanic %^a^	11.9%	34.0%	14.6%	20.1%	0.32 (0.04)
Education in years^a^, ^c^	15.50 (2.4)	16.71 (2.3)	15.96 (2.7)	16.82 (2.2)	0.02 (0.18)
Mini‐Mental State Examination^a^, ^c^	21.36 (5.3)	29.14 (1.0)	25.11 (3.3)	29.04 (1.2)	< 0.001 (0.85)
*APOE* ε4 carrier^b^, ^c^	55.6%	41.5%	65.6%	31.6%	0.007 (0.11)
CDR Global, % (0.5/1.0)	61.4%/38.6%	0.0%/0.0%	72.3%/27.7%	0.0%/0.0%	0.004 (0.12)
Cognitive status, % (MCI/ dementia)	26.2%/73.8%	0.0%/0.0%	53.5%/46.5%	0.0%/0.0%	< 0.001 (0.28)

*Note*: EOAD, early‐onset Alzheimer's disease; LEADS‐CN, cognitively normal participant from the Longitudinal Early‐onset Alzheimer's Disease Study; LOAD, late‐onset Alzheimer's disease; ADNI‐CN, cognitively normal participant from the Alzheimer's Disease Neuroimaging Initiative; CDR Global, Clinical Dementia Rating scale–Global score (range of 0, 0.5, 1.0, 2.0, 3.0). Values represent mean and SD unless otherwise noted. EOAD‐LOAD *p* values reflect results of independent samples *t*‐tests between EOAD and LOAD groups for continuous variables, and chi‐square or Fisher exact tests for categorical variables. Effect sizes were calculated using Cohen's *d* for continuous and Cohen's *w* for categorical variables.

^a^ Denotes significant difference between EOAD and CN‐LEADS groups, *p *< 0.001.

^b^ Denotes significant difference between EOAD and CN‐LEADS groups, *p *= 0.01.

^c^ Denotes significant difference between LOAD and CN‐ADNI groups, *p *< 0.001. Note, no CN to impairment group comparisons were conducted for CDR Global and Cognitive Status variables, as CN groups by definition had values of 0.

### 3.1 Cognitive profiles and primary analyses

**FIGURE 1:**
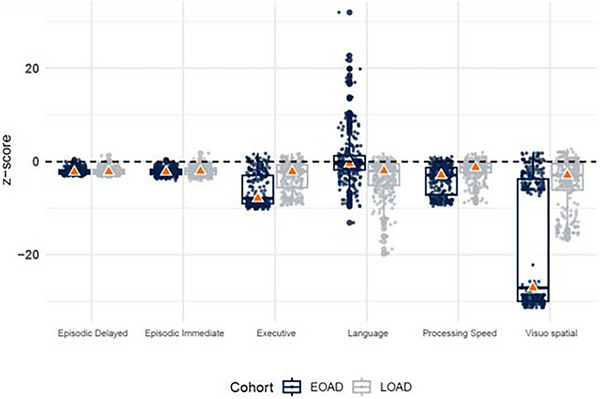
Box plots of domain scores by EOAD and LOAD participants. This figure shows the median domain score (triangle) for LOAD and EOAD participants, controlling for education, sex, *APOE* ε4 status, and MMSE performance. *APOE*, apolipoprotein E; EOAD, early‐onset Alzheimer's disease; LOAD, late‐onset Alzheimer's disease; MMSE, Mini‐Mental Status Examination.

Table 3 displays the median *robust Z‐*score performances for both EOAD and LOAD groups on the six cognitive domain composites, adjusted for education. As a reminder, a *Z‐*score reflects differences from expectation for that impairment group relative to the respective CN group; therefore lower (i.e., larger negative) *Z‐*scores between impairment groups reflect greater difference from expectation. Using a *Z‐*score of −1.5 or smaller to reflect clinical impairment (≥1.5 standard deviation (*SD*) below the median of the CN group), impairments were evident across nearly all domains for both EOAD and LOAD groups. The Visuospatial and Executive Functioning domains displayed the highest impairment relative to controls for the EOAD group, with impairment in Visuospatial and Episodic Delayed Memory domains being the largest for the LOAD group. Review of the distributional *Z‐*score plots in Figure 1 showed that performance for the EOAD group reflected a bimodal distribution for Visuospatial skills, with one portion of the sample performing comparably to the other cognitive domains and the other being notably impaired. The Language domain also displayed a high degree of variance within groups.

**TABLE 3:** Cognitive composite domain profiles of EOAD and LOAD groups.

	EOAD	LOAD	EOAD versus LOAD β (*SE*), *p* value, effect size
*N*	311	314	
Processing Speed/Attention	–2.94 (5.9)	–1.27 (2.0)	–0.87 (0.2), *p *< 0.001, 0.035
Episodic Delayed Memory	–2.25 (0.9)	–2.21 (1.0)	0.16 (0.1), *p = 0*.006, 0.013
Language	–0.47 (3.1)	–2.01 (4.6)	3.51 (0.4), *p *< 0.001, 0.105
Visuospatial Skills	–27.12 (26.2)	–2.94 (5.6)	–7.91 (0.8), *p *< 0.001, 0.151
Executive Functioning	–7.92 (6.1)	–2.17 (4.9)	–1.73 (0.3), *p *< 0.001, 0.067
Episodic Immediate Memory	–2.22 (1.1)	–2.08 (1.3)	0.24 (0.1), *p *< 0.001, 0.021

*Note*: EOAD and LOAD. Values represent median *Z*‐score composites (after accounting for education‐based residuals) and interquartile range unless otherwise noted. EOAD versus LOAD represent the beta coefficient (β), standard error (*SE*), *p* value, and effect size (*η^2^*) from the analyses comparing the respective groups after accounting for education, sex, *APOE ε4* status, and MMSE performance.

Abbreviations: *APOE*, apolipoprotein E; EOAD, early‐onset Alzheimer's disease; LOAD, late‐onset Alzheimer's disease; MMSE, Mini‐Mental State Examination.

In a between‐group comparison without accounting for the overall cognitive severity of the samples, Table 3 shows that the EOAD group generally performs worse than or equal to LOAD on most domains (*p’*s < 0.001 for Visuospatial, Executive Functioning, and Processing Speed/Attention; *p’*s > 0.05 for Language, Episodic Immediate Memory, and Episodic Delayed Memory). However, it is of note that Table 3 is only adjusted for education; therefore the relationships between groups for some domains change when accounting for these additional covariates. It is notable that after adjusting for the effects of sex, cognitive severity, and *APOE ε*4 carrier status in addition to education, we did not observe skewness and multimodality of the distributions; this suggests that we can assume conditional normality of the cognitive‐domain *Z‐*scores, and, therefore, that linear modeling is appropriate. Significantly lower Z‐scores were continued to be observed for EOAD relative to LOAD for Visuospatial Skills (*p *< 0.001; *η* ^2^ = 0.151), Executive Functioning (*p *< 0.001; *η* ^2^ = 0.067), and Processing Speed/Attention (*p *< 0.001; *η* ^2^ = 0.035), after accounting for education, sex, cognitive severity, and *APOE ε*4 carrier status. However, following added covariation (particularly cognitive severity), significantly lower Z‐scores were observed for LOAD relative to EOAD for Language (*p *< 0.001; *η* ^2^ = 0.105), Episodic Immediate Memory (*p *< 0.001; *η* ^2^ = 0.021), and Episodic Delayed Memory (*p = 0*.006; *η* ^2^ = 0.013). For the interested reader, raw score medians and interquartile ranges can be observed for each group in Table 4.

**TABLE 4:** Raw scores for individual cognitive measures among the study groups.

	CN‐ADNI	CN‐LEADS	EOAD	LOAD
RAVLT Total Recall	8.00 (6.0)	10.00 (5.0)	1.00 (2.0)	0.00 (2.0)
RAVLT Total Recall	45.00 (15.0)	48.00 (11.75)	20.00 (12.0)	26.00 (11.0)
Craft Story 21 Immediate Recall	15.00 (5.00)	16.00 (6.00)	6.00 (6.00)	6.00 (6.00)
Craft Story 21 Recall Delayed	13.00 (5.00)	15.00 (6.75)	3.00 (7.00)	3.00 (6.00)
Multilingual Naming Test	31.00 (2.0)	31.00 (3.0)	28.00 (6.0)	28.00 (6.0)
Animal Fluency	21.00 (7.0)	23.00 (6.0)	11.00 (7.0)	14.00 (7.0)
ADAS‐Cog Word Recognition^*^	11.00 (2.0)	12.00 (0.0)	12.00 (2.0)	6.00 (6.0)
ADAS‐Cog Naming Objects and Fingers^*^	5.00 (0.0)	0.00 (0.0)	0.00 (0.0)	5.00 (1.0)
ADAS‐Cog Commands^*^	5.00 (0.0)	0.00 (0.0)	0.00 (1.0)	5.00 (1.0)
MoCA Clock Numbers (% 0/1)	6.2%/93.8%	5.3%/87.2%	51.8%/45.0%	22.3%/77.4%
MOCA Clock Hands (% 0/1)	13.0%/87.0%	17.0%/75.5%	73.0%/23.8%	48.1%/51.6%
MoCA Cube (% 0/1)	25.7%/74.3%	28.7%/63.8%	74.6%/22.2%	51.0%/48.7%
Trail Making Test, Part A	30.00 (12.0)	24.00 (11.0)	61.00 (110.0)	44.00 (26.0)
Trail Making Test, Part B	67.0 (33)	59.0 (26.75)	300.0 (150.3)	126.0 (137.5)
ADAS‐Cog Cancellation	26.00 (8.0)	29.00 (5.8)	13.00 (12.0)	20.00 (8.0)

*Note*: ADAS‐Cog, Alzheimer's Disease Assessment Scale–Cognitive subscale; CN‐ADNI, cognitively normal group from the Alzheimer's Disease Neuroimaging Initiative; CN‐LEADS, cognitively normal group from the Longitudinal Early‐onset Alzheimer's Disease Study; EOAD, early‐onset Alzheimer's disease; LOAD, late‐onset Alzheimer's disease; RAVLT, Rey Auditory Verbal Learning Test. Values represent median raw scores and interquartile range unless otherwise noted.

* Denotes items being reverse scored for composite purposes.

### 3.2 Supplementary analyses

Sensitivity analyses revealed that removing EOAD cases with clinical phenotypes of frontal‐variant AD, PCA, and lv‐PPA (total *n *= 52) did not appreciably alter the results. Significantly lower Z‐scores continued to be observed for EOAD relative to LOAD for Visuospatial Skills (*p *< 0.001; *η* ^2^ = 0.139), Executive Functioning (*p *< 0.001; *η* ^2^ = 0.055), and Processing Speed/Attention (*p *< 0.001; *η* ^2^ = 0.025), after accounting for education, sex, cognitive severity, and *APOE ε*4 carrier status. Lower Z‐scores continued for LOAD relative to EOAD for Language (*p *< 0.001; *η* ^2^ = 0.129) and Episodic Immediate Memory (*p *= 0.004; *η* ^2^ = 0.015). No difference was observed between groups for Episodic Delayed Memory (*p = 0*.20; *η* ^2^ = 0.003), which is different from the primary analyses and likely results from only amnestic‐predominant participants remaining in this updated EOAD group.

## 4 DISCUSSION

The current study results highlight similarities and critical differences in cognitive profiles between sporadic EOAD and LOAD cohorts from LEADS and ADNI, respectively. Consistent with our hypotheses, although both AD cohorts displayed worse cognitive performance than their respective peers across all domains assessed, EOAD participants were more impaired than their LOAD counterparts on Visuospatial Skills, Executive Functioning, and Processing Speed/Attention, after accounting for cognitive severity, *APOE ε*4 status, and demographics. These findings were observed in the entire EOAD sample and also after excluding those with non‐amnestic phenotypic variants, and are consistent with several studies in the literature suggesting that EOAD possesses diffuse impairment in non‐memory cognitive domains at the time of presentation. Specifically, worse executive functioning, visual perception, attention/learning, and praxis have been observed in EOAD relative to LOAD in several studies.^12^, ^13^, ^14^, ^15^ Conversely, we observed that LOAD participants were more impaired for Episodic Immediate and Delayed Memory and Language. This also corresponded with expectations, as the literature has suggested either equivalence or disproportionately worse memory performance for LOAD groups.^12^, ^13^, ^14^, ^15^

Although generally comparable to previously observed trends, these findings are important because of several critical differences between LEADS and prior EOAD literature. First, LEADS possesses a larger sample of sporadic EOAD participants (*n *= 311) than those included in the aforementioned studies (*n’*s = 20, 38, 89, and 193), and the catchment for LEADS permits greater geographic representation (18 sites, spanning 3 million+ square miles) relative to single‐center cohorts. Second, the current EOAD sample complies with more rigid diagnostic criteria for AD, including evidence of amyloid positivity on amyloid‐PET; other studies either incorporated amyloid status from cerebrospinal fluid^13^, ^14^, ^15^ or were conducted before amyloid was required for AD diagnosis.^12^ Third, the current findings were seen despite accounting for discrepancies in cognitive severity between EOAD and LOAD cohorts, which has generally not been done previously. This was especially important given the magnitude of MMSE‐value differences observed currently (21.4 vs 25.5 for EOAD vs LOAD). This covariation ensured that our results were associated with true domain‐specific differences in cognitive performance, and not a function of overarching discrepancies in global severity of the participant populations between studies. Because the primary goal of ADNI is detection of AD “at the earliest possible stage” (https://adni.loni.usc.edu/about/), the recruitment goals for ADNI tended to result in less‐severe participants being recruited relative to LEADS.

The predominance of non‐amnestic cognitive dysfunction in EOAD relative to LOAD is consistent with their respective spatial patterns of neurodegeneration. LOAD is characterized by early and prominent episodic memory deficits associated with hippocampal and medial temporal atrophy, and region‐specific tau deposition.^51^, ^52^, ^53^, ^54^, ^55^ Although executive dysfunction is commonly seen in LOAD, it in part may be mediated by temporo‐parietal structures.^56^ Conversely, the observed EOAD profile appears to be driven primarily by prominent atrophy in the caudal‐lateral temporal cortex, posterior cingulate and precuneus cortices, and inferior parietal lobule, with relative sparing of the medial temporal lobe.^20^ Similarly, patients with EOAD have shown greater overall amyloid deposition than patients with LOAD, along with cortical‐predominant patterns of regional tau deposition (middle‐frontal gyrus, superior temporal gyrus, primary motor cortex, angular gyrus).^57^, ^58^ Furthermore, greater hypometabolism has been shown in EOAD than LOAD in the cingulate cortices and precuneus region.^59^ These posterior regions have been linked to deficits in visuospatial integration, working memory, attention, and executive functioning seen in EOAD.^60^ A portion of the EOAD sample also presented with PCA, which could in part explain the bimodal distribution of the Visuospatial domain; in addition, this bimodal distribution is likely related to the binary scoring of the measures comprising the Visuospatial composite (MoCA Clock Hands, Clock Numbers, and Cube; Table 1). Although memory functioning can also be impaired in EOAD samples—as could be seen in our clinically‐impaired Z‐scores for memory in Table 3—this appears to be a product of compromise to larger functional memory networks and to working memory and executive processes,^56^ as compared to consolidation deficits originating in the medial temporal lobe. Consequently, the separate profiles observed between EOAD and LOAD at present highlight that the two conditions are not distinguished solely by age at onset. Despite both conditions being associated with elevated amyloid deposition, their cognitive profiles and neurodegenerative patterns are otherwise distinct, and suggest rather diverging clinical entities. These results highlight the need for better understanding of biomarker contributions toward clinical manifestation of EOAD and LOAD, especially given recent diagnostic criteria for AD that focus exclusively on biomarker status for classification.^1^, ^61^

A few surprising observations from Table 3 are noteworthy. The first is that language performance was more impaired in LOAD relative to EOAD, as the limited literature has tended to suggest either equivalence between groups^13^, ^15^, ^62^ or worse performance for EOAD^13^ on certain measures. However, a couple studies have supported this disproportionately worse language functioning in LOAD,^12^, ^13^ which is likely driven by the relatively preserved anterior‐temporal lobes in EOAD.^20^, ^63^ In addition, although LEADS conducted a thorough examination of language, the language measures in ADNI were limited, with those overlapping between batteries being predominantly naming and category fluency. These measures are non‐specific and can be caused by a host of mechanisms beyond language, including semantic memory^64^—which tends to be compromised in LOAD.^65^ Future investigation of EOAD performance in language‐measures specific to lv‐PPA—like repetition—may uncover different results than those currently observed.

A second observation is related to the notably‐large negative*Z*‐scores seen for domains of Visuospatial Skills, Language, and (to some extent) Executive Functioning, which are beyond those typically seen in clinical settings (e.g., *Z *= −27.12). This is likely a function of both the binary response format of several of the tests comprising these domains, along with the tendency for “nearly‐perfect” performance in normal samples. For the relevant measures, intact individuals displayed minimal variability in performance, which led to extremely large negative *Z*‐scores (and large SDs) in our AD samples, especially for those with profound deficits due to lv‐PPA or PCA.

Third, although the *Z*‐score for Episodic Delayed Memory in LOAD is more impaired than in EOAD, it is not the worst domain *Z*‐score within the group. Given that memory impairment is the hallmark of AD,^1^ this observation is likely related to *Z*‐scores being artificially restricted for that domain psychometrically. For example, as mean performance on the RAVLT Delayed Recall using clinical norms^66^ is low at older ages due to normal aging,^67^ the worst raw score possible (score = 0) for 75‐year‐old men equals a *Z*‐score of −2.0. Conversely, EOAD participants on memory tasks and LOAD participants on non‐amnestic domains display wider ranges between age‐related mean performance and the floor for the measures; therefore, larger negative *Z*‐scores are possible. Examination of raw scores on RAVLT Delayed Recall closely showed that 32% of the LOAD sample scored 0, 1, or 2 of 15, suggesting that memory impairment was present but that the current use of *Z*‐scores clouded its observation.

Some limitations to the study are relevant. First, as alluded to earlier, domain composites could only incorporate measures included in the ADNI battery. As such, domains of visual‐memory, language, executive functioning, and visuospatial skills—which were assessed in detail in LEADS—could not be examined comprehensively or at all, in the case of visual‐memory. Although this may be a cause for some caution as the domains of executive functioning and visuospatial skills currently displayed the greatest effects for EOAD, our current results mirror our previous work in EOAD when examining a more extensive collection of executive functioning and visuospatial tasks.^11^ Second, analyses did not adjust for age differences between impairment and respective CN groups because age was so highly integrated in EOAD–LOAD classifications. Although this may have some impact on the *Z*‐score comparisons, both groups were similarly older than their CN groups (2.5 vs 3.3 years, respectively), and normatively this would have led to minimal clinical impact. Future research will investigate this for further impact. Third, results can only speak to sporadic forms of EOAD, as participants with the autosomal‐dominant forms are excluded from study protocols. Fourth, although the diversity representation was similar for impairment groups between LEADS and ADNI (Table 2), such sample homogeneity across studies limits the generalizability of these findings. Broadening ethnic and racial diversity will be important for all multicenter AD studies. Finally, LEADS and ADNI used different processes for diagnosis: LEADS used consensus criteria and ADNI used algorithms based on MMSE, Logical Memory, and CDR. Although this discrepancy may have influenced findings, we feel that this influence is likely limited because of (1) similarities between ADNI and LEADS criteria for CN, (2) reclassification of amyloid positivity of ADNI cases to match LEADS, and (3) selection of only “late MCI” cases from ADNI due to published concern about ADNI criteria.^68^

## 5 CONCLUSION

Our results support the previous literature indicating that EOAD and LOAD possess distinct cognitive profiles. Visuospatial and executive dysfunction appear to be particularly pronounced in EOAD, whereas memory and language impairments are worse in LOAD. Future work within LEADS is underway to examine more closely the genetic, fluid biomarker, and imaging distinctions between EOAD and LOAD, although our findings hint that EOAD and LOAD may be somewhat distinct clinical entities despite sharing a common neuropathology. Given the common tendency for both patients and clinicians to focus selectively on memory complaints during clinical presentations, these findings also suggest that clinicians should be cognizant of non‐amnestic impairments in younger populations to ensure the proper identification and intervention using disease‐modifying treatments.

## CONFLICT OF INTEREST STATEMENT

No authors associated with this project have reported conflicts of interest that would impact these results. Author disclosures are available in the Supporting Information.

## CONSENT STATEMENT

All authors have read and provided consent to be associated with this manuscript.

## Supporting information

Supporting Information
